# The autism spectrum disorder risk gene *NEXMIF* over-synchronizes hippocampal CA1 network and alters neuronal coding

**DOI:** 10.3389/fnins.2023.1277501

**Published:** 2023-10-27

**Authors:** Rebecca A. Mount, Mohamed Athif, Margaret O’Connor, Amith Saligrama, Hua-an Tseng, Sudiksha Sridhar, Chengqian Zhou, Emma Bortz, Erynne San Antonio, Mark A. Kramer, Heng-Ye Man, Xue Han

**Affiliations:** ^1^Department of Biomedical Engineering, Boston University, Boston, MA, United States; ^2^Department of Biology, Boston University, Boston, MA, United States; ^3^Commonwealth School, Boston, MA, United States; ^4^Department of Mathematics, Boston University, Boston, MA, United States

**Keywords:** autism spectrum disorder, network analysis, E/I balance, functional connectivity, network closeness centrality, pairwise correlation, GCaMP6f

## Abstract

Mutations in autism spectrum disorder (ASD) risk genes disrupt neural network dynamics that ultimately lead to abnormal behavior. To understand how ASD-risk genes influence neural circuit computation during behavior, we analyzed the hippocampal network by performing large-scale cellular calcium imaging from hundreds of individual CA1 neurons simultaneously in transgenic mice with total knockout of the X-linked ASD-risk gene *NEXMIF* (neurite extension and migration factor). As *NEXMIF* knockout in mice led to profound learning and memory deficits, we examined the CA1 network during voluntary locomotion, a fundamental component of spatial memory. We found that *NEXMIF* knockout does not alter the overall excitability of individual neurons but exaggerates movement-related neuronal responses. To quantify network functional connectivity changes, we applied closeness centrality analysis from graph theory to our large-scale calcium imaging datasets, in addition to using the conventional pairwise correlation analysis. Closeness centrality analysis considers both the number of connections and the connection strength between neurons within a network. We found that in wild-type mice the CA1 network desynchronizes during locomotion, consistent with increased network information coding during active behavior. Upon *NEXMIF* knockout, CA1 network is over-synchronized regardless of behavioral state and fails to desynchronize during locomotion, highlighting how perturbations in ASD-implicated genes create abnormal network synchronization that could contribute to ASD-related behaviors.

## Introduction

Autism spectrum disorder (ASD) is a neurodevelopmental disorder that affects 1 in 36 children (by the age of 8) in the United States (Maenner et al., 2023). ASD is characterized by three core behavioral symptoms: impairments in communication, restrictive and repetitive behaviors, and difficulty with social interactions (American Psychiatric Association, 2013). As one of the most heritable neuropsychiatric disorders, the genetic basis of ASD are widely heterogeneous and often polygenic (Satterstrom et al., 2020). Human genomic studies have identified numerous genes implicated in ASD risk. To understand the contribution of these genes to ASD pathophysiology, transgenic mice (Crawley, 2012; Hulbert and Jiang, 2016) and non-human primates (Zhou et al., 2019) containing such gene disruptions have been developed to model aspects of the behavioral, molecular, and cellular phenotypes seen in individuals with ASD.

Many ASD risk genes are thought to disrupt neural circuit development, leading to elevated network excitability through increasing synaptic-level excitatory/inhibitory (E/I) balance (Gonçalves et al., 2013). While it is unclear how increased synaptic E/I ratio alters network dynamics in vivo, computational modeling has revealed that the E/I balance is critical for maintaining proper asynchrony within a network (Litwin-Kumar et al., 2011) and that an increased E/I ratio elevates neural synchrony (Litwin-Kumar and Doiron, 2012; Middleton et al., 2012). Thus, it has been hypothesized that ASD risk gene mutations over-synchronize neural networks, leading to a reduction in network information encoding that disturbs cognitive performance (Zohary et al., 1994; Cohen and Maunsell, 2009; Rubin et al., 2017). Consistent with this theoretical framework, ASD animal models with an increased E/I balance exhibit increased neuronal correlations, as well as deficits in social interaction (Yizhar et al., 2011; Selimbeyoglu et al., 2017) and sensory discrimination (Chen et al., 2020). While lacking single neuron resolution, EEG variability analysis in humans has allowed the estimation of neural synchrony. As EEG provides an aggregate measure of neural activity-dependent extracellular electrical currents, lower EEG variability is indicative of greater neural synchrony. One study showed that ASD individuals without detectable EEG epileptiform activity exhibited lower EEG variability and higher functional E/I ratios than typically developing children (Bruining et al., 2020). Lower EEG variability is associated with decreased accuracy on a facial recognition task in typically developing children (Mcintosh et al., 2008). Finally, a low-dose ketamine infusion in healthy adults, thought to increase the E/I ratio, creates specific deficits in a spatial working memory task (Murray et al., 2014). Together, these computational and experimental evidence, in both animal models and human subjects, indicate that E/I imbalance and neural synchrony contribute to ASD network pathophysiology which ultimately results in behavioral disruptions.

Mutations in an X-linked gene, *NEXMIF* (neurite extension and migration factor, also known as *KIDLIA*, *KIAA2022*, or *Xpn*) were first discovered in several males with ASD, intellectual disability, and other co-morbidities (Cantagrel et al., 2004; Van Maldergem et al., 2013). Since then, several studies have reported additional ASD individuals with mutations or deletions in the *NEXMIF* gene (Lim et al., 2013; Iossifov et al., 2014; Charzewska et al., 2015; Kuroda et al., 2015; De Lange et al., 2016; Farach and Northrup, 2016; Webster et al., 2017; Yuen et al., 2017; Lambert et al., 2018; Lorenzo et al., 2018; Panda et al., 2020; Stamberger et al., 2020; Wang et al., 2020). *NEXMIF* is now recognized as a Category 1 gene in the Simons Foundation Autism Research Initiative (SFARI) database, further implicating it as an ASD-risk gene. NEXMIF protein is expressed exclusively in neuronal nuclei and loss of *NEXMIF* expression leads to aberrant neuronal migration and reduced dendritic growth due to a dysregulation in actin dynamics in neurite tips (Gilbert and Man, 2016). Thus, *NEXMIF* is critical for proper dendritic extension and neuronal migration in the developing mouse cortex (Gilbert and Man, 2016). Additionally, *NEXMIF* knockdown results in a significant loss of synapses with a twofold greater loss of GABAergic synapses compared to glutamatergic synapses in cultured neurons (Gilbert et al., 2020), suggesting an increased synaptic E/I balance. *NEXMIF* knockout (NEXMIF KO) mice demonstrate a variety of behavioral deficits, most notably reduced social interaction, impaired communication vocalizations, and increased self-grooming (indicative of repetitive behavior).

We analyzed the publicly available atlas of gene expression in adult mice available from the Allen Brain Institute, and found that NEXMIF expression is the highest in the hippocampus (Allen Institute for Brain Science, 2004a; Hawrylycz et al., 2007). As hippocampal structure (Dager et al., 2007; Groen et al., 2010; Chaddad et al., 2017; English et al., 2017; Reinhardt et al., 2020) and function (Just et al., 2007; Green et al., 2013; Gu et al., 2015; Krach et al., 2015) are often disrupted in individuals with ASD, we examined the hippocampal network in NEXMIF KO mice to understand how ASD-implicated *NEXMIF* gene mutations alter hippocampal function at both the cellular and network levels. Because *NEXMIF* KO leads to profound learning and memory deficits (Gilbert et al., 2020), it is extremely difficult to train these animals on hippocampal-dependent learning and memory tasks. Thus, we examined how *NEXMIF* KO altered CA1 cellular dynamics and network connectivity patterns during locomotion, an important aspect of spatial memory, by performing cellular calcium imaging from tens to hundreds of individual CA1 neurons simultaneously in NEXMIF KO male mice and wild-type (WT) male littermates during locomotion. We found that KO of *NEXMIF* did not alter calcium event shape and frequency in individual neurons but increased behaviorally specific neuronal responses during locomotion. We then characterized network effects of *NEXMIF* KO using Pearson correlation and network closeness centrality and discovered that loss of *NEXMIF* creates over-synchronization of the CA1 network during locomotion.

## Results

### NEXMIF WT and KO mice exhibit similar locomotor behavior

**FIGURE 1:**
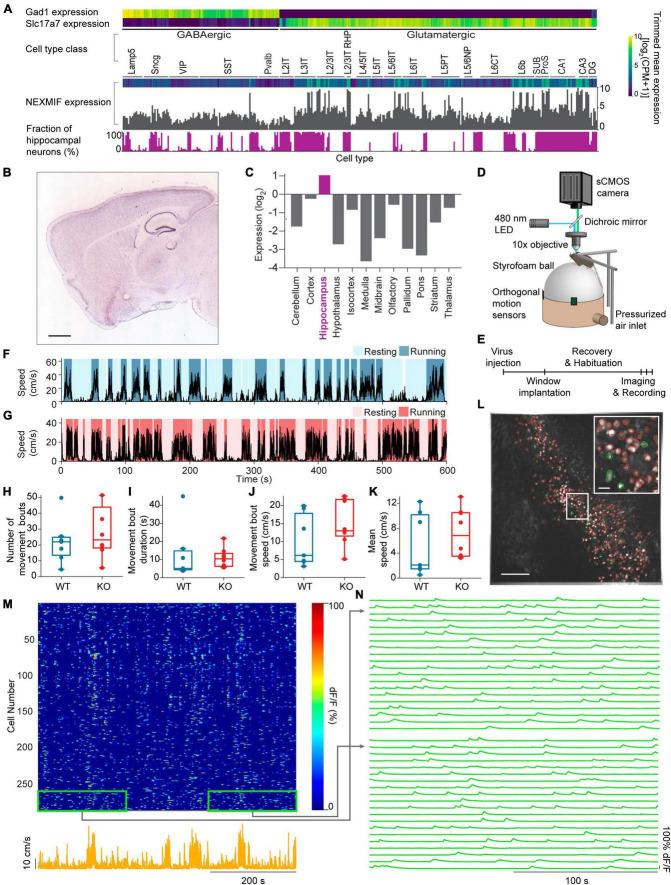
Experimental set-up and movement behavior. **(A)** Mouse cortex and hippocampus single cell RNA-Seq data from the Allen Brain Institute’s Cell Types Database showing expression levels for the GABAergic marker gene Gad1, the glutamatergic marker gene Slc17a7 and the *NEXMIF* gene in each transcriptomic cell type. Bottom, the fraction of hippocampal neurons among the total sequenced cells in each transcriptomic cell type. **(B)** RNA In-Situ Hybridization data showing *NEXMIF* expression in a sagittal slice including the hippocampus, from the Allen Brain Institute’s Mouse Brain Atlas, https://mouse.brain-map.org/experiment/show?id=69531127. Scale bar: 839 μm. **(C)** Quantification of relative expression levels of *NEXMIF* in panel **(B)**. **(D)** The experimental setup illustrating a mouse head-fixed under a custom wide-field microscope, voluntarily navigating a spherical treadmill. **(E)** Experimental timeline. Animal’s movement speed during an example experimental session in a WT animal **(F)** and a KO animal **(G)**. Identified resting (light blue and light pink) and running (dark blue and dark pink) bouts are overlaid on the movement speed traces. **(H)** Average number of movement bouts per 10-min session (WT: 22.0 ± 14.2 bouts, mean ± SD, *n* = 7 WT mice; KO: 28.4 ± 16.6 bouts, *n* = 8 KO mice, Wilcoxon rank sum test *p* = 0.44). **(I)** Average movement bouts duration (WT: 12.8 ± 14.9 s, KO: 10.9 ± 5.43 s, Wilcoxon rank sum test, *p* = 0.34). **(J)** Mean speed during movement bouts (WT: 10.16 ± 7.3 cm/s, KO: 15.04 ± 6.33 cm/s, Wilcoxon rank sum test, *p* = 0.19). **(K)** Average speed over the entire imaging session (WT: 5.36 ± 5.0 cm/s, KO: 7.25 ± 3.92 cm/s, Wilcoxon rank sum test, *p* = 0.34). Example maximum-minus-minimum projection fluorescence image across the entire recording session. All selected ROIs are outlined in red, with highlighted cells in panel **(L)** shown in green. Scale bar: 200 μm. Inset: zoom-in of white box. Scale bar: 40 μm. **(M)** Heat map of GCaMP6f dF/F traces for the ROIs shown in panel **(L)** during an example session (top) and animal’s corresponding movement speed (bottom). **(N)** Zoom-ins of the heat map regions outlined in green in panel **(M)**, showing the fluorescence traces for 20 representative cells from the beginning and the end of the imaging session. In panels **(H–K)**, each dot corresponds to an individual session (box: interquartile range, whiskers: 1.5 ± interquartile range, middle line: median).

Because of the various behavioral deficits observed in adult NEXMIF KO mice (Gilbert et al., 2020), we first examined NEXMIF expression profiles by analyzing the mouse cortex and hippocampus RNA-Seq data from the Allen Brain Institute’s Cell Types Database (Allen Institute for Brain Science, 2004b; Yao et al., 2021) and the RNA In-Situ Hybridization data from the Allen Brain Institute’s Mouse Brain Atlas (Allen Institute for Brain Science, 2004a; Hawrylycz et al., 2007). Interestingly, we found that NEXMIF expression is most prominent in the hippocampus (Figures 1B, C) without obvious difference between excitatory versus inhibitory neurons (Figure 1A), consistent with the observation that NEXMIF KO mice exhibit severe learning and memory deficits (Allen Institute for Brain Science, 2004a; Gilbert et al., 2020). To understand how *NEXMIF* contributes to hippocampal circuit functions, we then characterized CA1 neural responses using calcium imaging while mice were head-fixed and navigating freely on a spherical treadmill (Figure 1D). Since it is difficult for NEXMIF KO mice to perform hippocampal-dependent learning and memory tasks as observed in our previous study (Gilbert et al., 2020), we examined how *NEXMIF* KO changes hippocampal circuity during locomotion, a fundamental component of spatial memory.

We performed wide-field calcium imaging from hundreds of individual dorsal CA1 neurons simultaneously in both WT and KO animals during voluntary locomotion, comparing homozygous NEXMIF KO male mice with complete deletion of *NEXMIF* and their WT male littermates. Since *NEXMIF* is an X-linked gene and NEXMIF KO male mice are infertile, homogenous female mice cannot be generated. Thus, we used only male KO mice that have a complete deletion of *NEXMIF*. Briefly, we first injected AAV-Synapsin-GCaMP6f into the CA1 to label neurons specifically, and then surgically removed the overlying cortex and implanted an imaging window above CA1. The imaging field of view was centered on the stratum pyramidale, about 100 μm below the imaging window, though it is possible some interneurons in the stratum oriens were in the field of view as well. Each mouse was recorded for 10 minutes per day every other day over a 5-day period (Figure 1E). We did not detect noticeable differences across the three calcium imaging sessions from the same mouse, and thus all recording sessions from each mouse were grouped for further analysis.

We first examined voluntary movement kinematics between KO mice (*n* = 8 mice) and WT littermates (*n* = 7 mice). “Resting” and “running” bouts were identified based on movement speed (details in section “Materials and methods,” Figures 1F, G) simultaneously recorded with each imaging session. WT and KO mice exhibited a similar number of running bouts (periods of continuous running) within each 10-minutes session (Figure 1H), with similar running bout duration (Figure 1I) and speed (Figure 1J), and overall speed across the entire session (Figure 1K). Furthermore, these movement kinematic measures were not correlated with the age of the mice in either WT or KO groups (Supplementary Figure 1). Thus, *NEXMIF* KO does not alter overall movement kinematics in our experimental setting, allowing us to examine NEXMIF-induced changes in neuronal responses independent of behavioral alterations.

### Calcium event shape and frequency are undisturbed in NEXMIF KO mice

**FIGURE 2:**
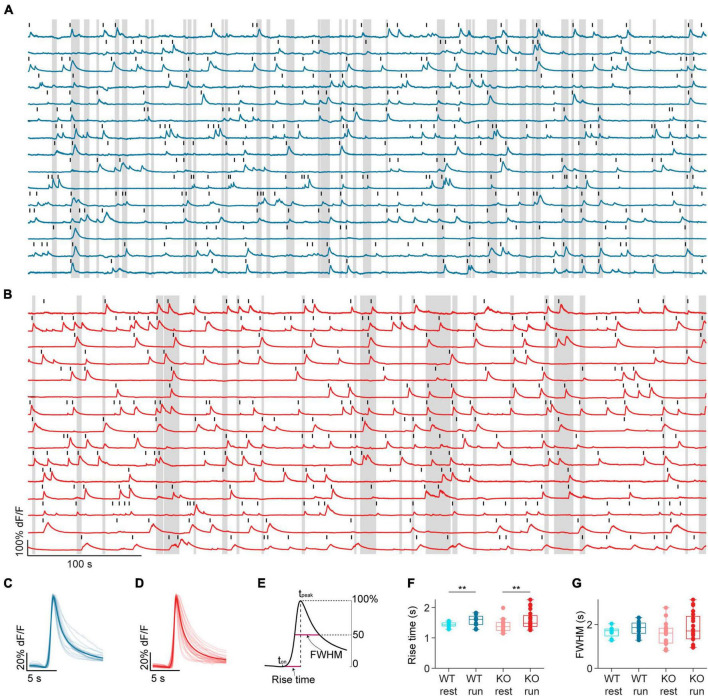
*NEXMIF* knockout does not change calcium event shape. Example fluorescence traces from **(A)** a WT animal and **(B)** a KO animal, with movement bouts shown as gray shading. Fifteen cells are shown from each mouse. Each detected calcium event is marked with a black line. Average calcium event shape from WT sessions (**C**, dark blue) and KO sessions (**D**, dark red). Events were first averaged within each cell, and then averaged across all cells in a session. Each session average is shown as a light line, and the population average is shown as the solid line. **(E)** Schematic calcium event rise time and full width at half-maximum (FWHM) calculation. **(F)** Mean calcium event rise time in WT mice during rest (light blue) and run (dark blue), and in KO during rest (light red) and run (dark red). (WT rest: 1.43 ± 0.08 s, mean ± SD, *n* = 12 sessions from 6 mice, WT run: 1.57 ± 0.19 s, KO rest: 1.40 ± 0.20 s, *n* = 20 sessions from 8 mice; KO run: 1.59 ± 0.30 s, Linear Model, behavioral condition: *p* = 0.008, WT/KO genotype: *p* = 0.71, interaction: *p* = 0.66.) **(G)** Mean FWHM in WT mice during rest (light blue) and run (dark blue), and in KO during rest (light red) and run (dark red). (WT rest: 1.65 ± 0.23 s, WT run: 1.84 ± 0.33 s, KO rest 1.56 ± 0.51 s, KO run: 1.86 ± 0.33 s, Linear Model, significance against intercept-only model: *p* = 0.21.) In panels **(F,G)**, each dot corresponds to an individual session (box: interquartile range, whiskers: 1.5 × interquartile range, middle line: median). **p < 0.01.

We next examined calcium events across individual neurons recorded in WT versus KO mice. The recorded calcium fluorescence videos were first motion corrected and individual cells were segmented (Shen et al., 2018; Figure 1L). A GCaMP6 fluorescence trace was then extracted for each cell and normalized to its peak fluorescence to account for variation in GCaMP6f expression between neurons (Figures 1M, N). We then identified individual calcium events as described previously (Zemel et al., 2022) (see section “Materials and methods,” Figures 2A–D). The identified calcium events occurred at a rate of 2.24 ± 0.50 events/min over the entire imaging session, similar to previous CA1 recordings using GCaMP6 (Mount et al., 2021), and there was no difference between WT and KO mice (WT: 2.10 ± 0.35 events/min, mean ± standard deviation (SD), *n* = 18 sessions from 7 mice; KO: 2.32 ± 0.53 events/min, *n* = 24 sessions from 8 mice, Wilcoxon rank sum test, *p* = 0.12). Thus, GCaMP6 calcium imaging is capable of capturing neural activity dynamics in both mice groups.

We estimated neural activity using both the rise time and the frequency of individual calcium events, as the rising phase of calcium events captures the sharp increases in intracellular calcium that are common during spike bursts (Huang et al., 2021). We found that calcium event rise time was longer during running than resting in both WT and KO mice, but there was no difference between KO and WT during either behavioral condition (Figure 2F). Additionally, we calculated full width at half-maximum amplitude (FWHM) to estimate calcium buffering capacity, as the duration of a calcium event captures overall intracellular calcium change (McMahon and Jackson, 2018; Huang et al., 2021). FWHM was similar regardless of behavioral condition or genotype (Figure 2G). Thus, *NEXMIF* KO does not affect the overall activity or calcium buffering capacity of CA1 neurons.

### *NEXMIF* KO increases the fraction of movement-modulated neurons

**FIGURE 3:**
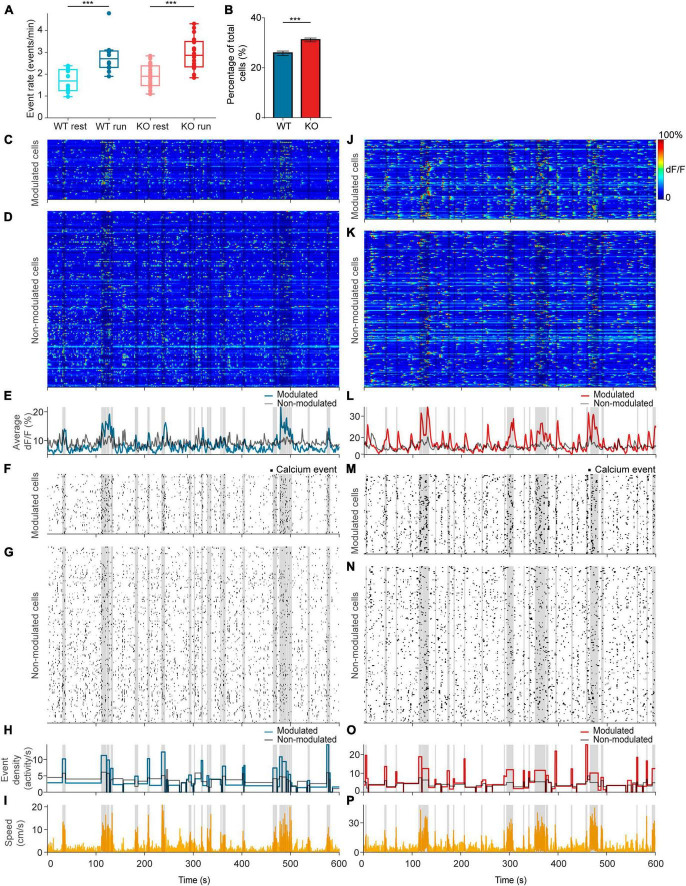
*NEXMIF* knockout increases fraction of movement-modulated cells. **(A)** Calcium event rate in WT mice during rest (light blue) versus run (dark blue), and in KO during rest (light red) versus run (dark red). (WT rest: 1.71 ± 0.51 events/min, mean ± SD, *n* = 12 sessions in 6 mice; WT run: 2.79 ± 0.75 events/min; KO rest: 1.90 ± 0.54 events/min, *n* = 20 sessions in 8 mice; KO run: 2.93 ± 0.75 events/min, Linear Model, behavioral condition: *p* = 4.91 × 10^–6^, WT/KO genotype: *p* = 0.41, interaction: *p* = 0.86.) Each dot corresponds to an individual session (box: interquartile range, whiskers: 1.5 × interquartile range, middle line: median). **(B)** Fraction of all neurons that are movement modulated in WT (blue) versus KO (red) mice (WT: 25.7 ± 2.01%, proportion ± 95% confidence interval, *n* = 1,805 cells from 6 mice; KO: 30.1 ± 1.8%, *n* = 2,530 cells from 8 mice, Fisher’s exact test, *p* = 1.6 × 10^–4^). Example sessions from a **(C–I)** WT animal and a **(J–P)** KO animal. **(C,J)** Heat map of GCaMP6f dF/F traces for movement-modulated cells and **(D,K)** non-movement-modulated cells in WT and KO mice. Average dF/F across movement-modulated cells in WT (**E**, blue) and KO (**L**, red), and non-movement-modulated cells (**E,L**, black). **(F,M)** Binarized calcium traces for all movement-modulated cells and **(G,N)** all non-movement-modulated cells in the example sessions. Average calcium event density across all movement-modulated cells in WT (**H**, blue) and KO (**O**, red), and non-movement-modulated cells (**H,O**, black). **(I,P)** Corresponding movement speed (orange) for the session. All plots are overlaid with movement bouts in gray. ***p < 0.001.

Since CA1 neurons are known to increase their activity during locomotion (Vanderwolf, 1969; Fuhrmann et al., 2015), we next compared calcium event rates during resting versus running. We found that calcium event rate across the entire population increased from resting to running in both WT and KO animals, but there was no difference between WT and KO (Figure 3A). After observing this population-level change in neural activity during locomotion, we next evaluated how individual CA1 neurons are modulated by movement. To determine whether a neuron is modulated by movement, we binarized the GCaMP6f dF/F trace (Figures 3C, D, J, K) to calcium event trace with ones assigned to the entire rising phase of each calcium event and zeros everywhere else (Figures 3F, G, M, N). We then computed the difference in event density during running versus resting and compared it to a shuffled null distribution. In each shuffle, we circularly shifted each binarized calcium trace by a random temporal offset relative to movement and calculated the difference in activity between the running periods and resting periods (Figures 3I, P). This procedure was repeated 1,000 times to form the null distribution. A cell was deemed to be movement-modulated if the observed neural activity difference was greater than the 97.5th percentile of the shuffled null distribution for that cell. Using this analysis, we found that 31.0% of neurons were movement-modulated in KO animals, significantly higher than the 25.7% observed in WT (Figure 3B). As expected, the movement-modulated cell population increased total dF/F during running, whereas the non-modulated cell population showed no difference between behavioral conditions (Figures 3E, L). Accordingly, event rate increased during running in the movement-modulated cell population, but did not change in non-modulated cells (Figures 3H, O). The percentage of cells that were movement-modulated in each session did not depend on the time the animal spent running or the animal’s average speed during the session for either mouse group or behavioral condition (Supplementary Figure 2). This increase in the proportion of movement-modulated cells in KO mice suggests that *NEXMIF* KO increases behavioral responses of the CA1 circuit. As *NEXMIF* KO increases E/I synaptic ratio of individual cells (Gilbert et al., 2020), our results support the hypothesis that increased synaptic level E/I ratio by ASD risk gene mutation increases behaviorally evoked network responses, consistent with the observation that sensory stimuli lead to an over-activation of the hippocampus in individuals with ASD (Green et al., 2013).

### *NEXMIF* KO increases functional connectivity between neuron pairs during running

**FIGURE 4:**
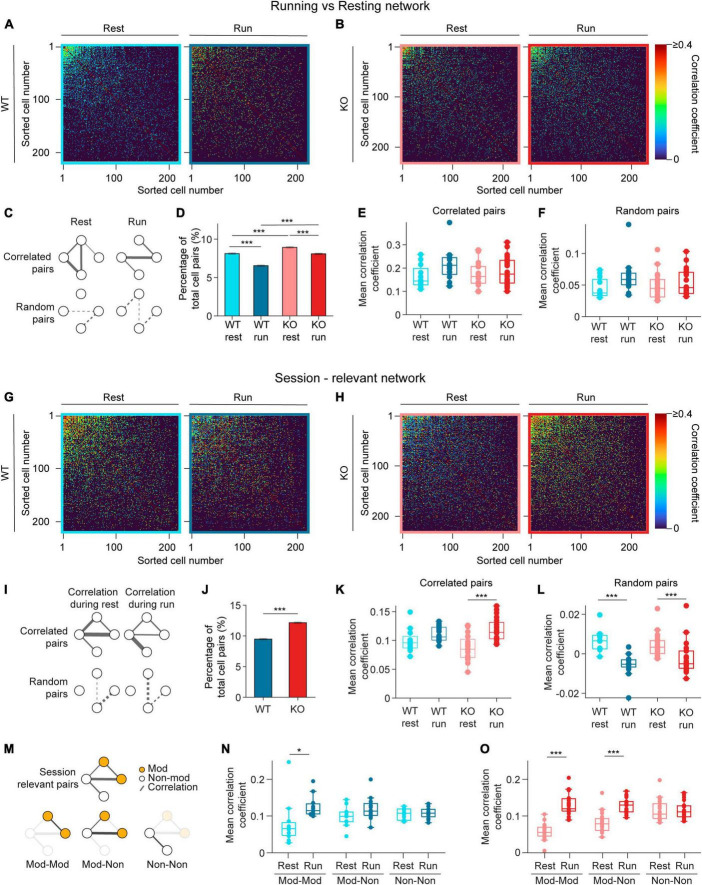
Pairwise correlation increases during running in NEXMIF KO mice. **(A)** Correlation matrices of pairwise Pearson correlation coefficient during resting (left) and running (right) for rest-relevant (left) and run-relevant neurons (right) from an example WT animal. Within each matrix, the most correlated cell pairs are sorted to the top left corner. Correlated pairs are colored corresponding to their correlation coefficient, random pairs are colored black. **(B)** Same as in panel **(A)**, but for an example KO animal. **(C)** Schematic networks showing (top left) resting-relevant and (top right) running-relevant pairs, and (bottom) the corresponding random pairs. **(D)** Fraction of neuron pairs that are correlated in WT mice during rest (light blue) and run (dark blue), and in KO during rest (light red) and run (dark red). (WT rest: 8.12 ± 0.10%, proportion ± 95% confidence interval, 301,335 neuron pairs, WT run: 6.55 ± 0.09%; KO rest: 8.93 ± 0.09%, 361,687 neuron pairs, KO run: 8.09 ± 0.09%. Fisher’s exact test, WT rest vs. WT run: *p* = 2.1 × 10^–121^, KO rest vs. KO run: *p* = 1.76 × 10^–37^, WT rest vs. KO rest: *p* = 7.7 × 10^–32^, WT run vs. KO run: *p* = 3.4 × 10^–127^.) **(E)** Pearson correlation coefficients during resting for rest-relevant and during running for run-relevant cell pairs (WT rest: 0.16 ± 0.05, mean ± SD, *n* = 12 sessions in 6 WT mice, WT run: 0.22 ± 0.07; KO rest: 0.17 ± 0.05, *n* = 20 sessions in 8 KO mice, KO run: 0.19 ± 0.06, Linear Model, significance against intercept-only model: *p* = 0.10). **(F)** Same as panel **(E)**, for random cell pairs. (WT rest: 0.05 ± 0.02, mean ± SD, *n* = 12 sessions in 6 WT mice, WT run: 0.06 ± 0.03; KO rest: 0.05 ± 0.02, *n* = 20 sessions in 8 KO mice, KO run: 0.05 ± 0.02, Linear Model, significance against intercept-only model: *p* = 0.14.) **(G)** Same as in panel **(A)**, for session-relevant neurons from the same WT animal. **(H)** Same as in panel **(B)**, for session-relevant neurons from the same KO animal. **(I)** Schematic of a session-relevant network with line widths denoting the correlation strength of correlated pairs (top left) during rest and (top right) during running, and (bottom) the corresponding random pairs. **(J)** Fraction of neuron pairs that are correlated in WT mice (blue) and in KO (red) mice during the entire session (WT: 9.48 ± 0.10%, proportion ± 95% confidence interval, 301,335 neuron pairs, KO: 12.15 ± 0.11%, 361,687 neuron pairs; Fisher’s exact test, *p* = 4.1 × 10^–266^). **(K)** Pearson correlation coefficients during resting or running of session-relevant cell pairs. (WT rest: 0.10 ± 0.02, mean ± SD, *n* = 12 sessions in 6 WT mice, WT run: 0.11 ± 0.01; KO rest: 0.09 ± 0.02, *n* = 20 sessions in 8 KO mice, KO run: 0.12 ± 0.02, Linear Model, interaction: *p* = 0.04, post-hoc Linear Model, WT rest vs. WT run: *p* ± 0.12; KO rest vs. KO run: *p* = 2.33 × 10^–6^.) **(L)** Same as panel **(K)**, for random cell pairs. (WT rest: 6 × 10^–3^ ± 5 × 10^–3^, mean ± SD, *n* = 12 sessions in 6 WT mice, WT run: −6 × 10^–3^ ± 6 × 10^–3^; KO rest: 4 × 10^–3^ ± 6 × 10^–3^, *n* = 20 sessions in 8 KO mice, KO run: −2 × 10^–3^ ± 8 × 10^–3^, Linear Model, behavioral condition: *p* = 4.29 × 10^–3^, WT/KO genotype: *p* = 0.40, interaction: *p* = 0.10.) **(M)** Schematic of (top) the session-relevant network in I and (bottom) the same network decomposed into three sub-networks in which both cells are modulated (Mod-Mod, left), one modulated and one non-modulated (Mod-Non, middle), or both are non-modulated (Non-Non, right). Session-relevant pairs are connected by a line, with modulated cells in orange and non-modulated cells in white. **(N)** Mean correlation coefficient of the three session-relevant sub-networks in WT animals (Mod-Mod rest: 0.08 ± 0.03, mean ± SD, *n* = 12 sessions in 6 mice, Mod-Mod run: 0.13 ± 0.03; Mod-Non rest: 0.10 ± 0.03, Mon-Non run: 0.12 ± 0.03; Non-Non rest: 0.11 ± 0.02, Non-Non run: 0.11 ± 0.02; Linear Model, interaction between Mod-Mod network and Behavior: *p* = 3.11 × 10^–3^, *post-hoc* Linear Model, rest vs. run: *p* = 3.67 × 10^–2^.) **(O)** Same as in panel **(N)**, for KO animals (Mod-Mod rest: 0.05 ± 0.03, *n* = 20 sessions in 8 KO mice, Mod-Mod run: 0.13 ± 0.03; Mon-Non rest: 0.08 ± 0.03, Mon-Non run: 0.12 ± 0.02; Non-Non rest: 0.11 ± 0.03, Non-Non run: 0.11 ± 0.03, Linear Model, interaction between Mod-Mod network and Behavior: *p* = 1.44 × 10^–8^, *post-hoc* Linear Model, rest vs. run: *p* = 6.69 × 10^–10^; Linear Model, interaction between Mod-non network and Behavior: *p* = 3.0 × 10^–4^, *post-hoc* Linear Model, rest vs. run: *p* = 8.53 × 10^–7^). In panels **(E,F,K,L,N,O)**, each dot corresponds to an individual session (box: interquartile range, whiskers: 1.5 ± interquartile range, middle line: median). *p < 0.05, ***p < 0.001.

Computational studies have shown that increased synaptic E/I ratio increases network synchrony measured as population pairwise correlations, thus decreasing network information coding capability (Zohary et al., 1994; Litwin-Kumar and Doiron, 2012; Middleton et al., 2012). Additionally, several animal models with deletions of ASD risk genes exhibit increased neuronal correlations (Selimbeyoglu et al., 2017; Chen et al., 2020). As *NEXMIF* KO increases E/I ratio, like many other ASD risk gene mutations, we next examined whether *NEXMIF* KO influences CA1 network synchrony by calculating Pearson correlation between the binarized traces of simultaneously recorded neuron pairs (Figures 4A, B, G, H). The binarized traces include only the rising phase of calcium events to avoid overestimation of correlation due to the slow decay kinetics of GCaMP6f. To account for variations in event rate, we determined whether the measured correlation between each neuron pair was significantly greater than chance observation given the event rates of the neurons in the pair. To estimate chance observations, we shifted the binarized traces of two neurons relative to one another with a random time lag and obtained a shuffled Pearson correlation coefficient. We repeated this shuffling procedure 2,000 times to create a shuffled null distribution. If the observed correlation coefficient was greater than the 95th percentile of the shuffled null distribution, the neuron pair was deemed significantly correlated (correlated pair). If the observed correlation coefficient was below the 95th percentile of the shuffled distribution, the correlation was deemed non-significant (random pair) (Figure 4C).

As many neurons exhibited elevated event rate during running (Figure 3A), we first identified correlated pairs during running (running-relevant pairs) versus resting (resting-relevant pairs) separately to account for variation in event rates during these periods. Specifically, to identify running-relevant pairs, we only considered the calcium event traces from neuron pairs when animals were running. Similarly, for resting-relevant pairs, only data during resting was considered. We found that the fraction of pairs that are correlated during running is smaller than during resting in both WT and KO mice (Figure 4D), and KO animals contained more correlated cell pairs compared to WT mice during both resting and running (Figure 4D). When we compared correlation coefficients between correlated pairs, we found no difference between WT and KO mice during both resting and running (Figure 4E). The correlation coefficients of random pairs were also similar between WT and KO during both behavioral conditions (Figure 4F). Thus, running desynchronizes the overall CA1 neural network by reducing the fraction of functionally connected neurons without altering connectivity strength between neuron pairs in both WT and KO groups. *NEXMIF* KO increases CA1 synchronization by increasing the fraction of functionally connected neurons without altering the connectivity strength during either resting or running.

Since the running-relevant pairs and resting-relevant pairs are often not the same neuron pairs, we could not directly compare how connectivity changes relevant neuron pairs during resting versus running. Thus, we next identified correlated pairs using calcium event traces throughout the entire session (session-relevant pairs) (Figures 4G, H). To identify session-relevant pairs, we compared the observed correlation between a neuron pair to the shuffled distribution using the entire recording period (Figure 4I). We found that the fraction of session-relevant pairs was increased in *NEXMIF* KO (Figure 4J). Interestingly, in WT mice, the correlation strengths of these session-relevant pairs were slightly higher during running, but were not significantly different between resting and running, indicating that when an animal switches between the two behavioral states, the relevant CA1 network connectivity remains largely stable (Figure 4K). In KO mice, however, correlation strength among session-relevant cells is significantly higher during running than resting (Figure 4K). In contrast, random pairs decreased their correlation strength during running in both WT and KO animals (Figure 4L).

To further investigate whether movement-modulated cells contribute to the increase of correlation strength in KO during running, we separately examined the correlation strength between two movement-modulated cells, between a movement-modulated and a non-modulated cell, and between two non-modulated cells (Figure 4M). In WT mice, correlation coefficient is significantly different only between two movement-modulated cells (Figure 4N), which may contribute to the small but non-significant increase across all pairs as shown in Figure 4K. However, in KO mice, correlation coefficients between two modulated neurons, and between a modulated and a non-modulated neuron pair were both higher during running than resting (Figure 4O). Thus, the increase in correlation coefficients in KO mice during running is a result of connectivity strength involving movement-modulated cells.

Since running increases event rates, we next evaluated how event rate impacts Pearson correlation coefficient measures. Under the condition of very sparse event rates observed in our study (WT: 2.12 ± 1.36 events/min, mean ± SD, *n* = 1817 neurons from 12 sessions in 6 mice, KO: 2.35 ± 1.48 events/min, *n* = 2845 neurons from 20 sessions in 8 mice), Pearson correlation coefficients decreased as event rate increases (Supplementary Figure 3). Thus, as running increased event rates, the observed increase in correlation coefficients cannot be explained by increased activity of individual neurons. Together, these results demonstrate that *NEXMIF* KO leads to over-synchronization of the CA1 network, particularly during running, by increasing the strength of pairwise correlations and synchronizing a larger fraction of CA1 neurons.

### Overall network connectivity is exaggerated during locomotion in *NEXMIF* KO

**FIGURE 5:**
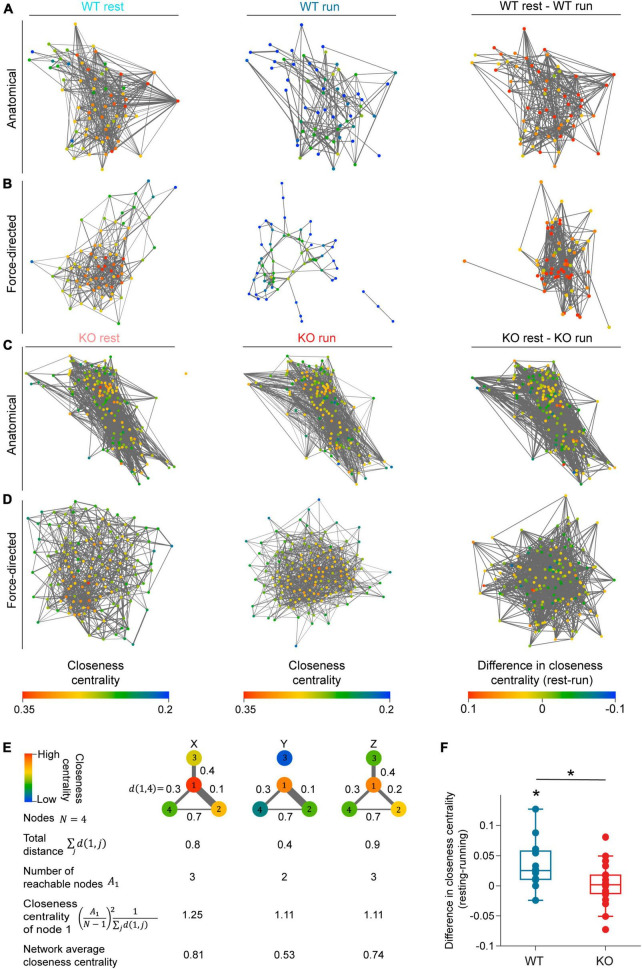
*NEXMIF* knockout increases overall functional connectivity of the CA1 network. Anatomical network maps of closeness centrality during (left) resting, (middle) running, and (right) the change between resting and running from example **(A)** WT and **(C)** KO animals. In left and middle columns, each cell is color coded based on its closeness centrality measure and correlated pairs in the corresponding behavioral condition are connected by an edge. In the right column, each cell is color coded based on the change in its closeness centrality from resting to running, and session-relevant pairs are connected by an edge. Edge width represents normalized correlation coefficient. **(B,D)** Similar to panels **(A,C)**, but shown as a force-directed graph. **(E)** A schematic of closeness centrality computation. An example network of four nodes is shown in three different network states (X, Y, Z) with the distance of the edge shown between each pair of nodes. Closeness centrality of node 1 in the three states are 1.25, 1.11, and 1.11, respectively. The reduction of node 1’s closeness centrality in state Y compared to state X is due to the loss of reachable node 3. The decrease of node 1’s closeness centrality in state Z relative to state X is due to the increased distance to node 2. **(F)** Average closeness centrality difference (resting-running) in WT (blue) and KO (red) mice. (WT: 0.036 ± 0.041, mean ± SD, *n* = 12 sessions from 6 mice, significantly greater than 0, Wilcoxon signed rank test, *p* = 6.8 × 10^–3^; KO: 0.002 ± 0.034, *n* = 20 sessions from 8 mice not significantly different from 0, Wilcoxon sign rank test, *p* = 0.77, Wilcoxon rank sum test between WT and KO, *p* = 0.02.) Each dot corresponds to an individual session (box: interquartile range, whiskers: 1.5 < interquartile range, middle line: median). *p < 0.05.

After establishing functional connectivity changes between neuron pairs using Pearson correlation, we further characterized connectivity of the CA1 network as a whole using graph theory analysis. We first created network maps using the correlated cell pairs during either resting or running. Each cell is a node in the map, and a correlated cell pair is connected by an edge between those two nodes. To quantify the connectivity of each network graph, we calculated the closeness centrality of each neuron. Closeness centrality is a metric used in graph theory to measure node importance, which takes both number of connections and connection strength (correlation coefficient) into account (details in section “Materials and methods”). Briefly, a greater closeness centrality value for a neuron indicates that the neuron is connected, both directly and indirectly, to a greater number of nodes in the network (Figure 5E).

As fluorescence imaging allowed us to visualize the anatomical relationship between recorded neurons, we first arranged the network graph using the anatomical position of each cell (Figures 5A, C). We did not observe any obvious spatial patterns in the closeness centrality within CA1 networks in the anatomical maps. Consequently, to better visualize the strength of network connectivity, we arranged each map as a force-directed graph where cells are positioned closer if their functional connectivity is higher regardless of their absolute anatomical location (Figures 5B, D). In WT force-directed maps, cells were more tightly clustered during resting than running, indicating higher connectivity during resting. However, KO force-directed maps showed similar amounts of clustering between resting and running (Figure 5B). We also noted that the change in each neuron’s closeness centrality varied widely from resting to running (Figures 5A–D). Thus, to quantify the changes in overall network connectivity between behavioral conditions, we computed the difference in average closeness centrality between the resting graph and running graph for each recording session (Figure 5F). We found that WT mice showed a significant decrease in closeness centrality during running compared to resting, demonstrating that the CA1 network is desynchronized during locomotion. This network-level observation is consistent with our pairwise Pearson correlation analysis showing that in WT mice, fewer cell pairs were correlated during locomotion while correlation strength remained constant (Figures 4D, K). In contrast, KO mice showed similar closeness centrality during running and resting, suggesting that KO network fails to desynchronize during locomotion (Figures 5C, D). This lack of overall network desynchronization in KO mice measured with closeness centrality could be due to the opposing effects we observed with Pearson correlation analysis, which showed the fraction of correlated cells in KO mice is lower during running (Figure 4D) while correlation strength is higher (Figure 4K). Further, WT mice exhibited a greater decrease in closeness centrality than KO mice, consistent with the higher fraction of correlated cells in KO mice compared to WT mice (Figure 4D). Together, these results confirm that while the WT CA1 network desynchronizes during locomotion, *NEXMIF* KO impairs CA1 network desynchronization.

## Discussion

In this study, we examined how loss of *NEXMIF*, an ASD risk gene highly expressed in the hippocampus, influences individual CA1 neurons’ responses and CA1 network functional connectivity using large-scale single-cell resolution calcium imaging. As NEXMIF KO mice exhibit profound learning and memory deficits as indicated by Barnes maze and novel object tests (Gilbert et al., 2020), we probed the hippocampal network during voluntary locomotion, a fundamental aspect of spatial memory. We compared the patterns of neural activation between NEXMIF KO and WT littermates during quiescent immobility versus active locomotion. We found that spontaneous calcium event rate is similar between WT and KO mice, but a larger percentage of CA1 neurons are activated during movement in KO mice. Furthermore, a greater fraction of neuron pairs is correlated in KO animals, and the KO network is overly synchronized during locomotion. Overall, our results demonstrate that loss of *NEXMIF* leads to increased behaviorally evoked responses and elevated network synchronization, both of which could contribute to the disruption of CA1 network coding ability during behavior.

Our previous work has shown that in an open-field task, NEXMIF KO mice are more active than WT mice, reflecting higher levels of anxiety in NEXMIF KO mice (Gilbert et al., 2020). We did not find a difference in locomotion kinematics between KO and WT mice, likely due to differences in experimental conditions. However, the average running speed observed is comparable to those reported previously. Further, locomotion behavior did not vary with the age of the mice. Thus, our experimental paradigm allows us to probe the impact of *NEXMIF* KO on neural circuits during locomotion in the absence of behavioral changes.

As increased cellular and synaptic level E/I ratio in ASD can lead to increased neuronal excitability, epilepsy occurs in about 10% of people with ASD (Lukmanji et al., 2019) [about 15 times higher than incidence in the general population (Fiest et al., 2017)] and is particularly prevalent in individuals with *NEXMIF* mutations (Tye et al., 2019; Stamberger et al., 2020). We did not observe differences in basal calcium event rate in NEXMIF KO mice, but we detected significantly more neurons that selectively increased their activity during movement in KO animals. These observations provide evidence that in *NEXMIF* KO conditions, the elevated synaptic E/I ratio is not correlated to a broad increase in spontaneous neuronal activity, but rather a selective increase in responding during relevant behavior. This behavioral state-specific increase in neuronal activity in the CA1 could be due to a global increase in synaptic inputs to the CA1 during movement, in which an increased E/I synaptic ratio leads to a greater excitatory drive to CA1 neurons. However, it is also possible that the observed increase in neuronal activity reflects movement-dependent changes in intrinsic neuronal excitability, in addition to altered synaptic inputs.

Another leading hypothesis of ASD pathophysiology argues for overconnectivity within local brain regions and underconnectivity between interconnected brain regions, supported by several exciting human studies (Casanova et al., 2002; Casanova, 2004; Girgis et al., 2007; Kennedy and Courchesne, 2008; Wang et al., 2013; Zikopoulos and Barbas, 2013; Di Martino et al., 2014). We observed increased fractions of functionally correlated CA1 neuron pairs in *NEXMIF* KO animals during both immobility and active locomotion, as well as increased correlation strength during running in session-relevant cell pairs from KO mice, particularly with higher contribution from movement-modulated cells. Additionally, while the WT network was desynchronized during running, the KO network failed to desynchronize. Interestingly, we also detected a reduction in correlation coefficients across random pairs during running in both WT and KO, consistent with an overall network desynchronization effect, even though the correlation coefficients between random pairs were not deemed significantly higher than chance observation given their event rate. Each of these observations is consistent with abnormally increased functional connectivity within the CA1 circuit of NEXMIF KO mice during locomotion, supporting the local overconnectivity hypothesis. The elevated E/I synaptic ratio could contribute to this increased functional connectivity (Litwin-Kumar and Doiron, 2012; Middleton et al., 2012), but again, we cannot rule out the possibility that *NEXMIF* KO also changes intrinsic biophysical properties that lead to the observed over-synchronization. While CA1 pyramidal cells are known to have limited lateral connections, the elevated E/I synaptic ratio could result from reduced inhibitory inputs from local interneurons or increased excitatory inputs from upstream areas. Further work is needed to better understand the exact mechanisms by which *NEXMIF* alters both cellular biophysical properties such as ion channel expression and functional connectivity between the hippocampus and its interconnected areas. Additionally, intracellular calcium signaling is known to be important for neuronal morphogenesis and migration during development. While this study is limited to adult animals, future studies using similar calcium imaging approaches during development could provide insights into how changes in intracellular calcium dynamics upon *NEXMIF* KO may influence neurite extension and migration and contribute to the connectivity changes observed here.

We probed network functional connectivity using two measures, Pearson correlation between pairs of neurons and network closeness centrality. In WT animals, the number of correlated cell pairs decreased during locomotion while correlation strength of session-relevant correlated pairs was stable, ultimately resulting in decreased closeness centrality of the WT network during running. These results indicate decreased functional connectivity in the WT CA1 network during movement. Such network desynchronization would lead to increased information encoding capability, consistent with the idea that the CA1 network encodes relevant information during active movement (Colgin, 2013). As locomotion is a fundamental component of spatial navigation and memory, this dynamic change in information coding capability would allow for flexible and efficient encoding of a WT animal’s current environment for spatial memory.

In NEXMIF KO animals, however, a larger number of cell pairs are significantly correlated in both behavioral conditions than in WT animals, and session-relevant cell pairs are dominated by stronger correlations during running. Additionally, network closeness centrality of the KO CA1 network failed to decrease during movement, in sharp contrast to the reduction seen in WT networks. These different measures all support the consequence of *NEXMIF* KO in exaggerating network synchrony and preventing network desynchronization during active behavior.

Our previous study revealed that loss of NEXMIF led to a reduction in mature functional spines, leading to reduced excitatory synaptic strength in NEXMIF KO mice. While there was a reduction of both glutamatergic and GABAergic synaptic proteins in KO mice, the reduction in GABAergic synaptic density was double the loss of glutamatergic synapses in cultured neurons (Gilbert et al., 2020). This increase in synaptic E/I ratio in KO mice (Gilbert et al., 2020) likely contributes to the observed network over-synchronization in KO mice, which would lead to a decreased information encoding capacity in the CA1 network of NEXMIF KO mice. The higher percentage of movement-modulated cells observed in KO mice could reflect a compensatory mechanism in the CA1, to homeostatically increase information encoding capability throughout development. Alternatively, this higher percentage could be due to the increased number of correlated cells during running, as these correlations could arise from common inputs to these cell pairs that are activated upon movement. Overall, our observations of increased functional connectivity indicate a reduced ability to process spatial information and spatial encoding that could lead to the impaired spatial memory and contextual fear memory observed in NEXMIF KO mice (Gilbert et al., 2020).

## Materials and methods

### Animal surgery and recovery

All animal procedures were approved by the Boston University Institutional Animal Care and Use Committee. Eight homozygous *NEXMIF* KO (maintained on a C57Bl/6 genetic background) male mice and seven WT male littermates were used in this study (Gilbert et al., 2020). Mice were 7–34 weeks old at the start of experiments. Animals first underwent stereotaxic viral injection surgery, targeting the hippocampus (anterior/posterior: −2.0 mm, medial/lateral: +1.4 mm, dorsal/ventral: −1.6 mm from bregma). Mice were injected with 500–750 nl of AAV9-synapsin-GCaMP6f.WPRE.SV40 virus, obtained from the University of Pennsylvania Vector Core (titer ∼6e12 GC/ml). Injections were performed with a blunt 33-gauge stainless steel needle (NF33BL-2, World Precision Instruments) and a 10 μl microinjection syringe (Nanofil, World Precision Instruments), using a microinjector pump (UMP3 UltraMicroPump, World Precision Instruments). The needle was lowered over 1 min and remained in place for 1 min before infusion. The rate of infusion was 50 nl/min. After infusion, the needle remained in place for 7–10 min before being withdrawn over 1 min. The skin was then sutured closed with a tissue adhesive (Vetbond, 3M). After complete recovery (7+ days after virus injection), animals underwent a second surgery to implant a sterilized custom imaging cannula (outer diameter: 3.17 mm, inner diameter: 2.36 mm, height: 2 mm). The imaging cannula was fitted with a circular coverslip (size 0, outer diameter: 3 mm, Deckgläser Cover Glasses, Warner Instruments), adhered to the bottom using a UV-curable optical adhesive (Norland Optical Adhesive 60, P/N 6001, Norland Products). During surgery, an approximately 3.2 mm craniotomy was created (centered at anterior/posterior: −2.0 mm, medial/lateral: +1.7 mm) and the cortical tissue overlaying the hippocampus was aspirated away to expose the corpus callosum. The corpus callosum was then thinned until the underlying CA1 became visible. The imaging cannula was then tightly fit over the hippocampus and sealed in place using a surgical silicone adhesive (Kwik-Sil, World Precision Instruments). The imaging window was secured in place using bone adhesive (C&B Metabond, Parkell) and dental cement (Stoelting). A custom aluminum head-plate was also affixed to the skull anterior to the imaging window. Analgesic was provided for at least 48 h after each surgery, and mice were single-housed after window implantation surgery to prevent damage to the head-plate and imaging window.

### Calcium imaging and movement data acquisition

After complete recovery from window implantation surgery (7+ days), animals were habituated to experimenter handling and head fixation on the spherical treadmill. Each animal was habituated to running on the spherical treadmill while head-fixed for at least 3 days prior to the first recording day. During each recording session, animals were positioned under a custom wide-field microscope and allowed to run freely on the spherical treadmill. The spherical treadmills consisted of a three-dimensional printed plastic housing and a Styrofoam ball supported by air (Dombeck et al., 2007). The imaging microscope was equipped with a scientific complementary metal oxide semiconductor (sCMOS) camera (ORCA-Flash4.0 LT Digital CMOS camera C11440-42U, Hamamatsu) and a 10 × 0.28 M Plan Apo objective (Mitutoyo). GCaMP6f excitation was accomplished with a 5 W light emitting diode (M470L4, ThorLabs). The microscope included an excitation filter (No. FF01-468/553-25, Semrock), a dichroic mirror (No. FF493/574-Di01-25 × 36, Semrock), and an emission filter (No. FF01-512/630-25, Semrock). The imaging field of view was 1.343 × 1.343 mm (1,024 × 1,024 pixels). Image acquisition was performed using HC Image Live (Hamamatsu), and images were stored offline as multi-page tagged image files (TIFs) for further analysis.

Each animal underwent three 10-min recording sessions, one per day, every other day over 5 days (Figure 1E). A total of 21 recording sessions were collected from 8 KO mice and 16 sessions were collected from 7 WT mice. In 24 recording sessions (from 4 WT mice and 8 KO mice), a custom MATLAB script was used to trigger image frame capture at 20 Hz and to synchronize image acquisition with movement tracking. Digital transistor-transistor logic (TTL) pulses were delivered to the camera via a common input/output interface (No. USB-6259, National Instruments), and TTL pulses were also recorded using a commercial system (RZ5D, Tucker Davis Technologies). Motion data was collected using a modified ViRMEn system (Gritton et al., 2019). Movement was tracked using two computer universal serial bus mouse sensors affixed to the plastic housing at the equator of the Styrofoam ball, 78° apart. The mouse sensors’ x- and y-surface displacement data were acquired at 100 Hz on a separate computer, and a multi-threaded Python script was used to send packaged <dx, dy> data to the image acquisition computer via a RS232 serial link. Packaged motion data was recorded on the image acquisition computer using a modified ViRMEn MATLAB script and synchronized to each acquired imaging frame.

In the remaining 13 sessions (from 4 WT mice and 2 KO mice), image acquisition was triggered using a Teensy microcontroller system (Romano et al., 2019), and experiments were performed using an identical spherical treadmill. Digital pulses were sent from a Teensy 3.2 (TEENSY32, PJRC) to the sCMOS camera via SMA connectors and coaxial cables to trigger frame capture at 20 Hz. TTL pulses were recorded using the same TDT commercial system. Movement was tracked using two computer mouse sensors (ADNS-9800 laser motion sensors, Tindie) affixed to the plastic housing at the equator of the Styrofoam ball, about 75° apart. The x- and y-surface displacement was collected by the Teensy at 20 Hz and sent to the image acquisition computer via a standard USB-microUSB cable.

### Movement analysis

As both movement data acquisition systems collect the same numerical data, linear velocity can be calculated the same way for all sessions. Linear velocity in perpendicular *X* and *Y* directions was calculated as:


$$X=\frac{L-R\,c\,o\,s\,\theta }{\text{cos}\,\theta \,\left(\frac{\pi }{2}-\theta \right)}$$

Y=R

where *L* is the vertical reading from the left sensor, *R* is the vertical reading from the right sensor, and θ is the angle between the sensors. Linear velocity *V* was then calculated as:


$$V=\sqrt{X^{2}+Y^{2}}$$

Linear velocity values were then interpolated at 20 Hz.

To identify sustained periods of movement with high linear velocity (running bouts), we used a Fuzzy logic-based thresholding algorithm. We first assigned each velocity data point a Fuzzy membership value using a sigmoidal membership function *F*:


$$F\,\left(V,a,c\right)=\frac{1}{1+e^{-a\,\left(V-c\right)}}$$

where the threshold *c* is the 20th percentile of the velocity of that session or 5 cm/s, whichever is higher. *a* is set at 0.8. The resulting velocity trace was then smoothed using a moving average filter of 1.5 s. Next, the smoothed trace was thresholded at 10% of its maximum value. Time periods with velocity higher than this threshold that were at least 2 s long were considered high velocity periods (“running”). Time periods with velocity lower than this threshold that were at least 2 s long were considered low velocity periods (“resting”). Periods that did not satisfy either of these requirements were not considered for locomotion analysis (Figures 1F, G). Recording sessions in which the mouse spent less than 60 s (10% of the session) in one behavioral condition and sessions with less than 5 running bouts were not included for locomotion-related analysis (4 sessions from 2 WT mice and 1 session from 1 KO mouse).

### Calcium imaging video motion correction

Videos were first motion corrected using a custom Python script as described previously (Keaveney et al., 2020). For each imaging session, we first generated a reference image by calculating the mean value of each pixel across the first 2,047 frames. We then performed a series of contrast-enhancing procedures to highlight image features as follows. We used a Gaussian filter (Python SciPy package, ndimage.Gaussian_filter, sigma = 50) to remove the low-frequency component, which represents the potential non-uniform background. We then captured the edges of the high-intensity area by calculating the differences between two Gaussian filtered images (sigma = 2 and 1). To obtain the edge-enhanced image, the edges were multiplied by 100 and added back to the first filtered image (sigma = 2). Finally, to prevent a potential overall intensity shift caused by photobleaching, we normalized the intensity of each image by subtracting the mean intensity of the image from each pixel and dividing by the SD of the intensity. We then calculated the cross-correlations between the processed reference image and each processed image frame, and obtained the displacement between the peak of the coefficient and the center of the image. We then applied a horizontal shift, opposite to the displacement, to the original frame to finalize the motion correction.

### Cell segmentation

From the motion corrected video, a projection image was generated across all frames by subtracting the minimum fluorescence from the maximum fluorescence of each pixel (max-min projection image), and regions of interest (ROIs) corresponding to fluorescent cell bodies were automatically identified in the max-min projection image using a deep-learning algorithm based on U-Net (Ronneberger et al., 2015; Falk et al., 2019; Xiao et al., 2020). We first trained the deep-learning algorithm with manually curated data, containing the datasets reported in our previous studies (Shen et al., 2018; Zemel et al., 2022). For each training dataset, a max-min projection image was calculated as described above. We then divided the projection images and their corresponding ROI masks into small patches of 32 × 32 pixels as our training dataset. We also normalized each patch by shifting its mean intensity to zero and dividing the intensity of each pixel by the SD of the patch intensity. During training, each pixel was further augmented by randomly flipping vertically and/or horizontally, and rotating 90°C, 180°C, or 270°C.

To segment ROIs for the datasets in this study, the max-min projection image for each dataset was divided into 32 × 32 patches with 50% of each patch overlapping with its neighboring patches. Each patch was normalized as described above. As a result, each pixel was inferred four times, and we averaged the results from four inferences as the prediction score. The connected pixels with a predication score >0.5 were segmented as a potential ROI, and the set of segmentations was further refined with watershed transformation to obtain the ROIs representing single neurons. ROIs were then overlaid on the max-min projection image and manually inspected. ROIs that were identified by the machine learning algorithm but were not identified as a cell by the observer were manually removed. ROIs were then manually added to select cells that the machine learning algorithm did not properly identify. ROIs were added as a circle with a radius of 6 pixels (7.8 μm) based on morphology present in the max-min projection image, using the previously reported semi-automated custom MATLAB software called SemiSeg^1^ (Mount et al., 2021).

### GCaMP6f fluorescence trace extraction and normalization

We obtained the GCaMP6f fluorescence for each cell as the average fluorescence intensity across all pixels in that ROI. We then subtracted background fluorescence from each ROI, where the background fluorescence is the average pixel intensity across the pixels located within a ring centered at the corresponding cell ROI with an outer radius of 50 pixels and an inner radius of 15 pixels. The areas corresponding to other cell ROIs were excluded from this background ROI. Because the motion correction procedure introduces strips with high pixel intensities along the edges of the max-min projection image, 25 pixels along each edge of the image were also excluded from the calculation of background fluorescence. The resulting fluorescence trace for each cell was then interpolated at 20 Hz, linearly detrended (MATLAB function detrend), and normalized between 0 and 1. All traces were then manually inspected. Traces with large artifacts were removed.

### Calcium event detection

Onsets of calcium events were identified in each fluorescence trace similarly to previous descriptions (Mount et al., 2021; Zemel et al., 2022). Briefly, we first applied a moving average filter of 1 s to smoothen each trace and calculated the spectrogram [MATLAB chronux, mtspecgramc with tapers = (2, 3) and window = (1, 0.05)], and averaged the power below 2 Hz. We then calculated the change in power at each time point (powerdiff) and identified outliers (3 median absolute deviations away from the median power) in powerdiff (MATLAB function isoutlier) to detect all significant changes in trace power. When multiple outliers occurred at consecutive time points, they were classified as a potential calcium event. We then calculated the rise time and amplitude (the difference in fluorescence value between the peak and the event onset) for each potential event and used an iterative process to include only true events and exclude incorrect potential events. Within each iteration, an amplitude threshold was calculated for each potential event [iteration 1: 7 SDs of the trace in the 10 s (200 data points) prior to calcium event onset]. Potential events with a rise time greater than 150 ms (3 data points) and an amplitude above the calculated threshold were marked as correctly identified events for analysis. All the data points corresponding to these events (from beginning of event rise to end of event fall) were removed prior to the next iteration. We then repeated this process by re-calculating the amplitude threshold for the remaining potential events and again marking correctly identified events for analysis using the same criterion for rise time and the new amplitude threshold. For each successive iteration, the amplitude threshold was decreased by 40% and the duration to inspect prior to calcium event onset was increased by 75%. The iterative process stops once no events are marked as correctly identified events. This iterative method is more robust in capturing events that occur close together, while only minimally increasing identification of false positives. The preceding event in a sequence will incorrectly bias the SD of the trace in the window prior to a following event in the sequence, and this bias is removed when the preceding event is not included in the window prior to the event onset. All traces were then manually inspected to confirm event detection accuracy.

### Calcium event parameter and event rate analysis

For each detected calcium event, the rise time is defined as the duration from the calcium event onset, t_on_ to its peak t_peak_ (Figure 2E). To determine the full width at half-maximum amplitude (FWHM), we first calculated event height as the fluorescence different between t_on_ and t_peak_. FWHM was determined as the duration between the two points at 50% of the event height (Supplementary Figure 4A). If a subsequent calcium event was detected before the end point of the FWHM, the given event was excluded from FWHM analysis (Supplementary Figure 4B). Because it is difficult to reliably estimate FWHM if an event contains multiple small peaks, we further examined the number of peaks above 75% of the event height, and if more than one peak was identified (Supplementary Figure 4C), the given event was also excluded from FWHM analysis.

Total event rate was calculated across the entirety of each trace, counting each identified calcium event as one event. Event rate during either running or resting was calculated by counting the number of calcium events in all bouts of the relevant behavioral condition and dividing by the total time that the mouse spent in that behavioral condition.

### Determination of movement-modulated cells

To determine movement-modulated cells, we binarized each fluorescence trace by assigning ones to the entire rising phase (t_on_ to t_peak_) of each calcium event and zeros to the rest of the trace. We then concatenated all of the resting or running bouts separately, and summated the binarized trace among each concatenated period (“total activity”). We then subtracted the total activity during resting from the total activity during running to create an activity metric A. The calculation can be summarized as:


$$A=\left(\frac{\sum_{r\,u\,n}x}{\sum_{r\,u\,n}t}-\frac{\sum_{r\,e\,s\,t}x}{\sum_{r\,e\,s\,t}t}\right)\times 100\%$$

where *x* is the binarized calcium trace, and *t* is time. Next, we created a shuffled distribution of the activity metric for each cell by circularly shifting the binarized trace relative to the movement trace by a uniformly distributed random time lag 1,000 times and calculating *A* for each shuffle. If the true (non-shifted) *A* for a neuron was greater than the 97.5th percentile of the shuffled distribution, the cell was considered movement-modulated. Cells that did not meet this criterion were considered non-movement-modulated.

### Pairwise Pearson correlation analysis

For pairwise correlation analysis, we calculated the Pearson correlation coefficient between the binarized traces for each pair of neurons. Each binarized trace was the same as that used in determination of movement-modulated cells [ones to the entire rising phase (t_on_ to t_peak_) of each calcium event and zeros to the rest of the trace]. Only neuron pairs that were at least 20 pixels (26.2 μm) apart were included in all correlation analysis to eliminate potential fluorescence cross-contamination. We calculated pairwise correlation during resting alone, during running alone, or during the entire duration of the session. To calculate pairwise correlation during resting alone or running alone, we concatenated the calcium activity during all resting or running periods. To calculate pairwise correlation during the entire duration of the session, we used the full length of the calcium traces for each cell pair.

To determine whether the correlation coefficient for each cell pair was above chance level for each behavioral condition, we created a shuffled distribution of correlation by circularly shifting one trace relative to the other trace by a uniformly distributed random time lag 2,000 times and calculating the Pearson correlation coefficient for each shuffle. If the empirical (non-shifted) Pearson correlation for a pair of neurons was greater than the 95th percentile of the shuffled distribution, the cell pair was considered correlated. Positive correlation coefficients between neuron pairs that were not greater than the 95th percentile were not considered significant (random pairs). Negative correlations were not included in any analyses due to the sparseness of GCaMP6f events.

To estimate connectivity among modulated cells, we calculated the number of correlated movement-modulated cell pairs as a fraction of all movement-modulated cell pairs. Similarly, we also calculated the number of correlated non-movement-modulated cell pairs as a fraction of all non-movement-modulated cell pairs.

### Relationship between Pearson correlation coefficient and event rate analysis

As the calcium event rates detected in our study were sparse, we investigated the relationship between event rate and Pearson correlation coefficient. Specifically, to determine whether increasing event rate leads to an increase in pairwise Pearson correlation coefficient values by chance, we examined the relationship of the mean event rate of a neuron pair versus their shuffled correlation coefficient values. To calculate the shuffled correlation coefficient values of a neuron pair, we circularly shifted the calcium event vector of one neuron relative to the other by a random time lag uniformly distributed over the entire length of the trace, so that the temporal structure between the calcium event rates of the two neurons was destroyed. Next, Pearson correlation coefficient was calculated between the shuffled calcium event vectors of the neuron pair. This procedure was repeated 100 times for each cell pair, using either resting or running periods separately for each imaging session. These shuffled correlation coefficient values were then plotted against the mean event rate of the pair (Supplementary Figure 3A). The observed (true) correlation coefficients of session-relevant correlated pairs (Supplementary Figure 3B) and random pairs (Supplementary Figure 3C) were similarly plotted against the average event rate of each pair of neurons.

### Network closeness centrality analysis

To quantify network connectivity patterns, we calculated closeness centrality similarly to the description in Wuchty and Stadler (2003). Specifically, for each session, we created an undirected graph using correlated cell pairs during running and an undirected graph using correlated cell pairs during resting. Each cell was considered as a node and each correlated cell pair was connected by an edge. Edge weight was the Pearson correlation coefficient (calculated in the appropriate state, rest or run) between the binarized calcium traces of the cell pair. For each node *i*, closeness centrality *c*(*i*) is defined as:


$$c\,\left(i\right)={\left(\frac{A_{i}}{N-1}\right)}^{2}\,\frac{1}{C_{i}}$$

where *A_i_* is the number of nodes reachable to node *i* and *C*_*i*_ is the sum of distances from node *i* to all reachable nodes. The distance *d*(*i*,*j*) between nodes *i* and *j* is defined as:


$$d\,\left(i,j\right)=\sqrt{\log\left(\frac{1}{w_{i,j}}\right)}$$

where *w*_*i*,*j*_ is the edge weight. Closeness centralities of all the nodes were averaged within each network and multiplied by the number of nodes for normalization across networks with different numbers of nodes. Force-directed networks were created using a MATLAB implementation of a force-directed node placement algorithm that spatially clusters nodes proportional to *d*(*i*,*j*) (Fruchterman and Reingold, 1991).

### Statistical analysis

Statistical analyses were performed using MATLAB. Using Shapiro-Wilk’s normality test, we determined that most of our datasets do not follow normal distribution. Thus, non-parametric tests were used. Specifically, Wilcoxon rank sum test was used for comparisons between two groups (Figures 1H–K, 5F) and Linear Models (LMs) were used for comparisons between three or more groups. LMs were used to test whether the independent variables (WT/KO genotype, rest/run behavioral conditions, or different types of cell groups) were significant predictors for the dependent variable *Y* of interest using the following models:

For Figures 2F, G, 3A, 4E, F, K, L:


Y∼1+g⁢e⁢n⁢o⁢t⁢y⁢p⁢e+b⁢e⁢h⁢a⁢v⁢i⁢o⁢r⁢a⁢l⁢c⁢o⁢n⁢d⁢i⁢t⁢i⁢o⁢n+g⁢e⁢n⁢o⁢t⁢y⁢p⁢e×b⁢e⁢h⁢a⁢v⁢i⁢o⁢r⁢a⁢l⁢c⁢o⁢n⁢d⁢i⁢t⁢i⁢o⁢n

For Figure 4N:


$$Y_{W\,T}∼1+b\,e\,h\,a\,v\,i\,o\,r\,a\,l\,c\,o\,n\,d\,i\,t\,i\,o\,n+c\,e\,l\,l\,g\,r\,o\,u\,p+b\,e\,h\,a\,v\,i\,o\,r\,a\,l\,c\,o\,n\,d\,i\,t\,i\,o\,n\times c\,e\,l\,l\,g\,r\,o\,u\,p$$

For Figure 4O:


$$Y_{K\,O}∼1+b\,e\,h\,a\,v\,i\,o\,r\,a\,l\,c\,o\,n\,d\,i\,t\,i\,o\,n+c\,e\,l\,l\,g\,r\,o\,u\,p+b\,e\,h\,a\,v\,i\,o\,r\,a\,l\,c\,o\,n\,d\,i\,t\,i\,o\,n\times c\,e\,l\,l\,g\,r\,o\,u\,p$$

Maximum likelihood estimation was used to estimate coefficients for the selected model. First, we compared the model’s fit against an intercept-only model using a deviance test. If the model was significantly different from the intercept-only model, p values were calculated for the coefficient of each independent variable (genotype, behavioral condition, and cell group), and the interaction term (genotype × behavioral condition or behavioral condition × cell group). If the coefficient of the interaction term was significant, separate post-hoc Linear Models were used to test whether behavioral condition (Figures 2F, G, 3A, 4E, F, K, L: *Y_WT_* ∼1 + *behavioral condition*, and *Y*_*KO*_ ∼ 1 + *behavioral condition*, Figure 4N: *Y_WT, cellgroup_* ∼ 1 + *behavioral condition*, Figure 4O: *Y_KO, cellgroup_*∼1 + *behavioral condition*) was a significant predictor of the dependent variable. If the interaction term was not significant, independent variables with significant coefficient terms (*p* < 0.05) were considered as the significant predictors of the dependent variable. Further, because the variables in our study (genotypes, cell groups, and behavioral conditions) have only two levels (WT vs. KO, rest vs. run, modulated versus non-modulated cell pairs), a post-hoc test was not required when interaction term was not significant. Wilcoxon signed rank tests were used to test if medians were significantly different from 0 (Figure 5F). Finally, when comparing proportions (Figures 3B, 4D, J), Fisher’s exact test was used. Error bars show the 95% confidence interval defined as follows.


$$C\,o\,n\,f\,i\,d\,e\,n\,c\,e\,I\,n\,t\,e\,r\,v\,a\,l=P\pm 1.96\,\sqrt{\frac{P\,\left(1-P\right)}{n}}$$

where *P* denotes the percentages, and *n* denotes the number of samples. Simple linear regression was used to compare the percentage of movement-modulated cells versus movement bout duration (Supplementary Figure 2A) or average speed (Supplementary Figure 2B), and kinematic measures vs. age (Supplementary Figures 1A–D).

## Data availability statement

The raw data supporting the conclusions of this article will be made available by the authors, without undue reservation.

## Ethics statement

The animal study was approved by the Boston University Institutional Animal Care and Use Committee. The study was conducted in accordance with the local legislation and institutional requirements.

## Author contributions

RM: Conceptualization, Data curation, Formal analysis, Investigation, Methodology, Resources, Software, Validation, Visualization, Writing—original draft, Writing—review and editing. MA: Data curation, Formal analysis, Methodology, Resources, Software, Validation, Visualization, Writing—review and editing. MO’C: Methodology, Resources, Writing—review and editing. AS: Methodology, Software, Writing—review and editing. H-AT: Methodology, Software, Writing—review and editing. SS: Methodology, Writing—review and editing. CZ: Methodology, Software, Writing—review and editing. MK: Methodology, Writing—review and editing. EB: Software, Writing—review and editing. EA: Software, Writing—review and editing. H-YM: Methodology, Writing—review and editing, Resources, Validation, Writing—review and editing. XH: Conceptualization, Funding acquisition, Methodology, Project administration, Resources, Supervision, Validation, Writing—review and editing.

## Footnotes

1
github.com/HanLabBU/SemiSeg
