# Supplementary material for: The autism spectrum disorder risk gene NEXMIF over-synchronizes hippocampal CA1 network and alters neuronal coding
Source: Front Neurosci. 2023 Oct 27;17:1277501. doi: 10.3389/fnins.2023.1277501 (PMC10641898; doi:10.3389/fnins.2023.1277501)
Supplement: Supplementary file 1 [file Data_Sheet_1.PDF]

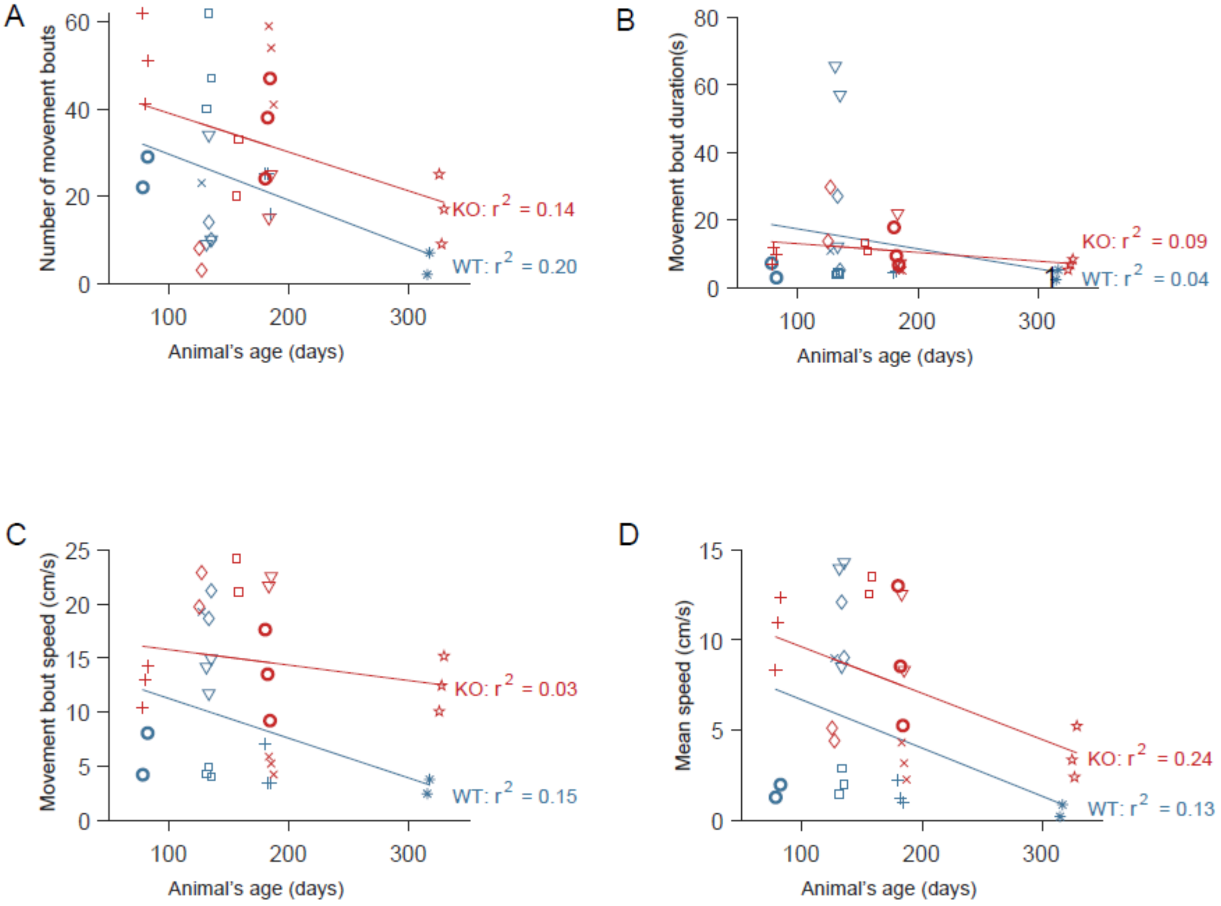


**Figure S1. Kinematic measures of locomotion are not correlated with the age of WT or KO mice.** (A) The number of movement bouts during each 10-minute session versus animal's age at the time of the recording. (Linear regression: WT: $p = 0.08$; KO: $p = 0.12$, $n = 16$ sessions from 7 WT mice and $n = 21$ sessions from 8 KO mice). (B) Mean movement bout duration versus animal's age at the time of each recording. (Linear regression: WT: $p = 0.44$; KO: $p = 0.22$) (C) Mean speed during movement bouts versus animal's age at the time of each recording. (Linear regression: WT: $p = 0.14$; KO: $p = 0.49$). (D) Mean speed over the entire imaging session versus animal's age at the time of each recording (Linear regression: WT: $p = 0.17$; KO: $p = 0.04$). Each marker shape represents a unique animal, red: KO and blue: WT. The lines indicate the corresponding linear regression fit across all sessions recorded in the KO (red) and WT (blue) animals, with the corresponding R-squared values.

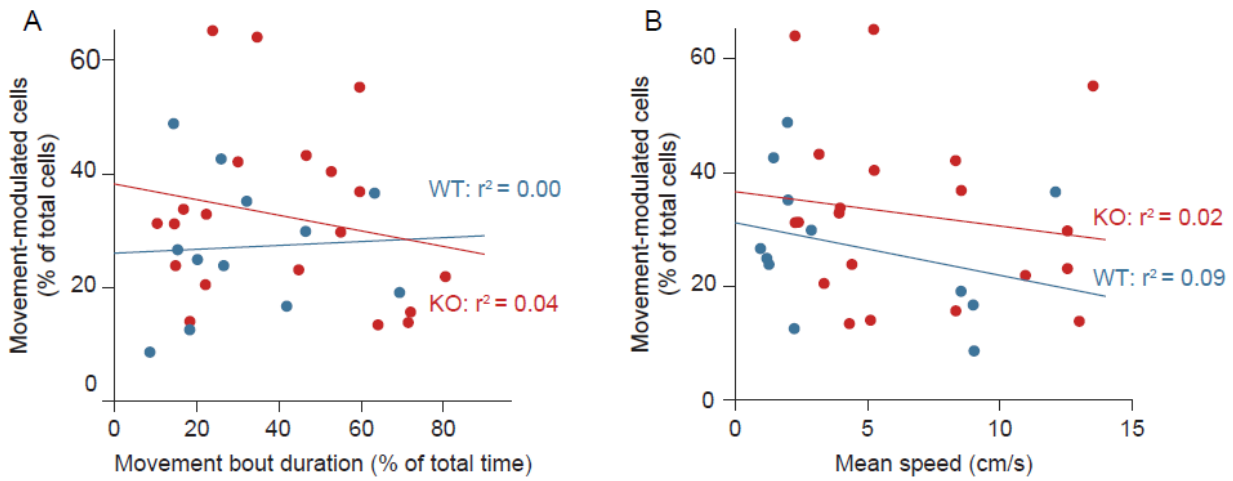


**Figure S2. Percentage of movement-modulated cells are not correlated with movement bout duration or running speed. (A)** Percentage of movement-modulated cells versus movement bout duration. (Linear regression: WT: p = 0.87, n = 12 sessions in 6 mice; KO: p = 0.40, n = 20 sessions in 8 mice). **(B)** Percentage of movement-modulated cells versus mean movement speed during the entire session. (Linear regression: WT: p = 0.33; KO p = 0.52). Each dot indicates a recording session, with red corresponding to KO and blue to WT animals. Linear regression is shown for each population with R-squared value.

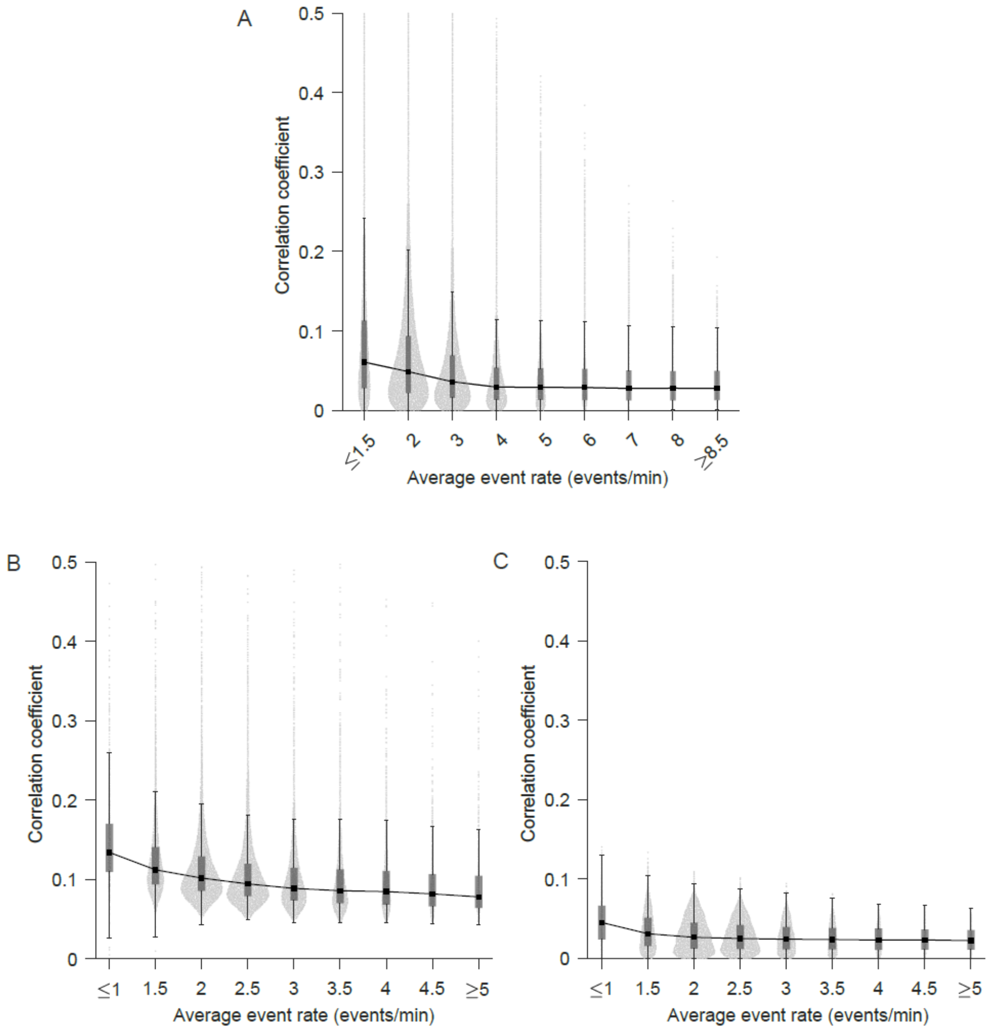


**Figure S3. When calcium event rate is low, pair-wise correlation coefficient decreases with increasing event rate. (A)** Correlation coefficient of temporally shifted traces versus the mean event rate of each neuron pair**.** Mean event rates are discretized into bins of 1 events/min. **(B, C)** Observed (true) correlation coefficient between neuron pairs versus the mean event rate of a that cell pair, for (B) correlated pairs and (C) random pairs. Mean event rates are discretized into bins of 0.5 events/min. Each dot corresponds to a pair of neurons. Individual data points with correlation coefficients beyond 0.5 are not shown in A and B. Each event rate bin is visualized as a violin plot (outer shape: data kernel density) and a boxplot (box: interquartile range, whiskers: 1.5 × interquartile range). The line plot connects the median of each bin.

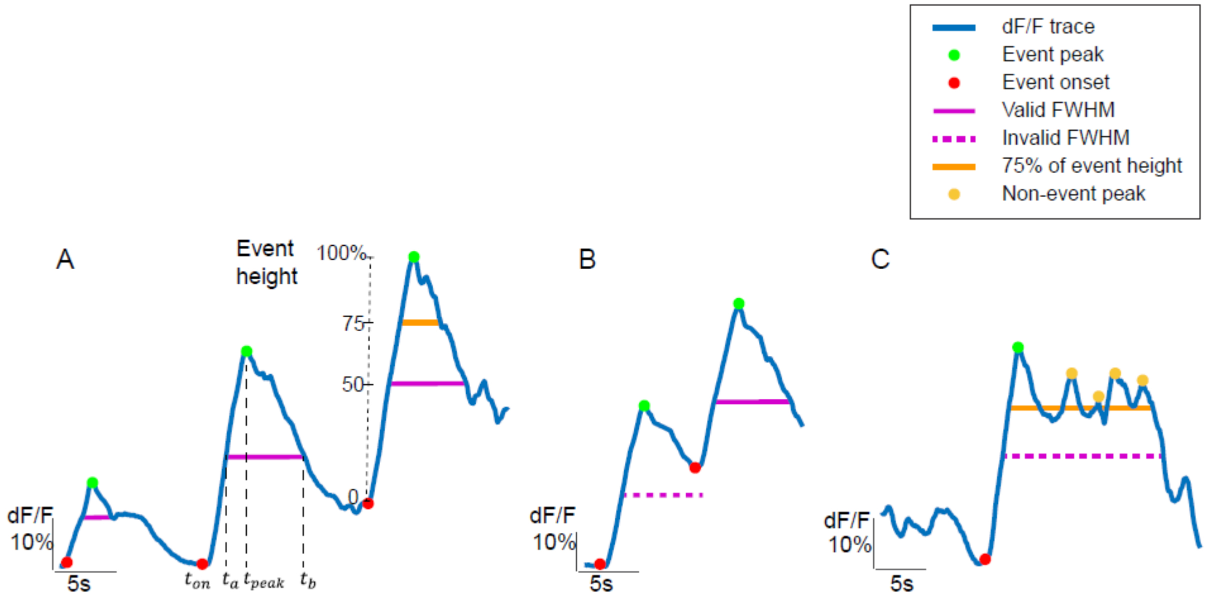


**Figure S4. Illustration of rise time and full width at half-maximum (FWHM) calculation. (A)** A sequence of events that are in close proximity, with each containing only one event above 75% of event height. Rise time is calculated as the interval between event onset (red dot) and event peak (green dot), and FWHM is calculated as the width at 50% of the event height. **(B)** An example where a second calcium event occurs before the end of the FWHM of the previous event. The first event is excluded from FWHM analysis. **(C)** An example event with more than one peaks above 75% of the event height. The rise time of the event is between the event onset and the event peak. But this event is excluded from FWHM analysis.
